# C-reactive protein-to-albumin ratio is associated with mortality after transcatheter tricuspid valve repair

**DOI:** 10.1007/s00392-025-02641-4

**Published:** 2025-04-10

**Authors:** Karl Finke, Laura Marx, Jan Althoff, Thorsten Gietzen, Matthieu Schäfer, Jan Wrobel, Philipp von Stein, Jennifer von Stein, Maria Isabel Körber, Stephan Baldus, Roman Pfister, Christos Iliadis

**Affiliations:** 1 Faculty of Medicine, Department III of Internal Medicine, University of Cologne, University Hospital Cologne, Cologne, Germany; 2 Cardiovascular Research Foundation, New York, United States

**Keywords:** Tricuspid regurgitation, Transcatheter tricuspid valve repair, Biomarker, Malnutrition, Inflammation, Right heart failure

## Abstract

**Background:**

Transcatheter tricuspid valve repair (TTVr) is a treatment option for tricuspid regurgitation (TR) in patients with high surgical risk. Given the heterogeneity in clinical benefit, there is a need for markers to assess mortality risk in patients undergoing TTVr. The C-reactive protein (CRP)/albumin ratio (CAR) is a marker of systemic inflammation and reduced nutritional status, which can both occur in TR.

**Methods:**

Consecutive patients undergoing TTVr at a tertiary care center were retrospectively analyzed. Serum CRP and albumin were collected at baseline. Intraprocedural success (IS) was defined according to TVARC criteria. The primary outcome of all-cause mortality was assessed up to 2 years after TTVr.

**Results:**

A total of 215 patients (69% females, median age 80 years) were identified. IS was achieved in 61% of patients. AUC of CAR for 2-year mortality was 0.695, with an optimal threshold of 1.2945 (Youden index) dividing patients in high CAR (*n* = 93) and low CAR (*n* = 122) groups. In the high CAR group, the primary endpoint occurred more frequently (43% vs 15%, *p* < 0.001) and significantly higher right atrial pressure, worse renal function, and less IS during TTVr were observed. High CAR was independently associated with an increased mortality risk even when adjusted for renal and liver function, right-ventricular function, and procedural failure (HR 2.188; 95%CI 1.2–3.9; *p* = 0.011).

**Conclusion:**

Higher CAR reflects patients with advanced right-heart failure and extracardiac organ damage and is associated with mortality after TTVr. CAR is derived from readily available parameters and may be useful additive to established risk scores.

**Graphical Abstract:**

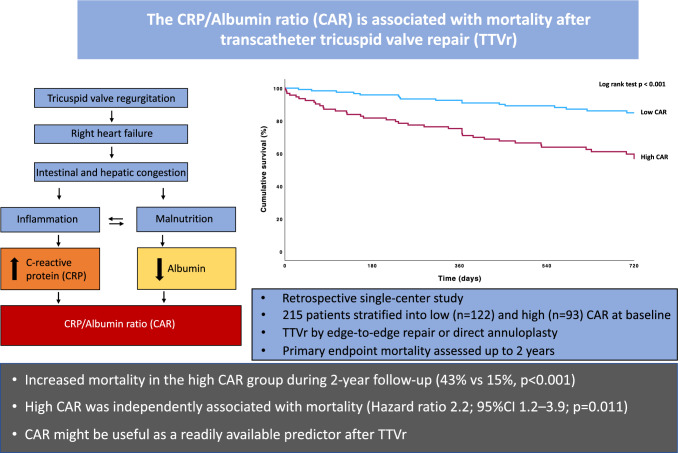

**Supplementary Information:**

The online version contains supplementary material available at 10.1007/s00392-025-02641-4.

## Introduction

Transcatheter tricuspid valve repair (TTVr) is increasingly recognized as a treatment option for severe tricuspid regurgitation (TR), due to the high safety profile and good efficacy for patients with high surgical risk [1]. TTVr can currently be performed through tricuspid valve transcatheter edge-to-edge repair (T-TEER) by leaflet approximation or transcatheter tricuspid valve annuloplasty (TTVA) [1]. TTVr has recently shown to improve patient reported quality of life, but significant improvement in mortality has yet to been shown [2]. Additionally, there is high heterogeneity in treatment benefit among patients treated with TTVr [3]. Patient selection and timing of transcatheter tricuspid valve repair remain a challenge as progression to right-ventricular (RV) failure with systemic impact might reduce the benefit of intervention, as patients gradually develop complications, ultimately culminating in multi-organ failure [4]. With increasing severity, TR causes elevated filling pressures and volume overload in the right heart, RV remodeling, and failure. This progressively leads to severe congestion, renal and liver impairment with decreased hepatic synthesis, as well as intestinal congestion causing low-grade systemic inflammation and cachexia by intestinal malabsorption with a pronounced impact on quality of life [5, 6]

While there are scores for mortality prediction in surgical candidates, data are lacking in the setting of transcatheter treatment. Patients undergoing transcatheter treatment are mostly elderly and frail, which leads to more complications [7, 8]. The surgery-derived TRI-SCORE, which consists of 12 parameters, has been associated with worse survival, but overestimates mortality after transcatheter treatment [9]. There is still a need for biological markers resembling patient factors, like low-grade inflammation, malnutrition, and organ damage, which could aid in the selection for TTVr [4, 5].

C-reactive protein (CRP) is produced by the liver and is elevated in tissue injury and infections, and is a marker of inflammation [10]. Albumin is also produced by the liver and is reduced in malnutrition, liver failure, and inflammation, and low levels are associated with frailty and cardiovascular disease [11].

The CRP/albumin ratio (CAR), as a marker of inflammation and malnutrition, has shown to predict mortality in critically ill patients including patients with septic shock [12], pancreatitis [13], acute kidney injury [14], and aortic stenosis, but has not yet been evaluated in patients undergoing tricuspid valve repair [15, 16]. Therefore, the aim of this study was to investigate CAR as a predictor of mortality after TTVr.

## Methods

### Study population and data collection

This study was conducted at the heart center of a German tertiary university hospital. The study protocol conformed to the 1975 Declaration of Helsinki and is in line with the established Ethical Guidelines for Epidemiological Research. All subjects provided informed consent prior to inclusion. We retrospectively studied 215 consecutive patients undergoing TTVr by T-TEER or TTVA from 2019 to 2022. T-TEER was performed using the TriClip™ (Abbott Vascular) or PASCAL (Edwards Lifesciences) systems. TTVA was performed with the Cardioband system (Edwards Lifesciences). Laboratory parameters were collected at baseline before procedure. The CAR was calculated by dividing CRP by the albumin level and the value was multiplied by 1000 for better readability. Echocardiographic parameters were assessed at baseline and postprocedural before discharge. Invasive hemodynamic measurements were performed directly before TTVr by right-heart catheterization. Clinical and demographic data were collected from the medical records. Intraprocedural success was defined according to Tricuspid Valve Academic Research Consortium (TVARC) criteria. [17] The EuroSCORE II and TRI-SCORE were calculated as described elsewhere [18, 19]. Standardized 6-min walk test (6-MWT) was performed at baseline and 30-day follow-up. Quality of life was assessed using the Minnesota Living with Heart Failure Questionnaire (MLHFQ) at baseline and 30-day follow-up. [20] Improvement of New York Heart Association (NYHA) functional class was defined as an improvement of at least one functional class. In general, all patients with sepsis were excluded from TTVr. Furthermore, all patients with clinical suspicion of infection prior to TTVr had blood cultures taken to exclude a systemic bacterial infection. To evaluate the role of infections in mortality, the outliers, which were cases above the 95th percentile of CRP values, were investigated separately.

### Primary endpoint

The primary endpoint of all-cause mortality after TTVr was assessed during the index hospital stay and up to 2 years after procedure. If the endpoint was not already met during follow-up visits, assessment of vital status was done by telephone contact with the patient or general practitioner.

### Statistical analysis

Normal distribution was tested for all variables using the Shapiro–Wilk test. Continuous variables were not normally distributed and are presented as median with interquartile range (IQR). Numbers and percentages were used to present nominal and ordinal data. Continuous variables were compared using the Mann–Whitney *U* test. Nominal data were tested for significant differences using the Pearson Chi-squared-test and the Fisher’s exact test, when appropriate.

Receiver-operating characteristic (ROC) analysis with area under the curve (AUC) was performed to evaluate the sensitivity and specify of CAR. Youden index was calculated to define an optimal cut-off to separate the overall population into a low and high CAR group.

Survival curves were created using Kaplan–Meier analysis and differences in survival were assessed using the log-rank test. The primary endpoint all-cause mortality and the secondary endpoint heart failure hospitalizations during follow-up were presented as a Kaplan–Meier estimates. Furthermore, we used Cox regression models to investigate the impact of CAR on mortality. Risk was expressed as hazard ratio (HR), 95% confidence interval (CI), and *p* value. Univariable Cox regression analysis was conducted for all baseline variables. Variables with a *p* < 0.05 in the univariable Cox regression analysis were selected for adjustment in a multivariable Cox regression model. Variables with multicollinearity were excluded from the regression analysis. Natriuretic peptides were not included in the regression analysis because of multiple confounders (renal dysfunction and obesity). A two-tailed *p* value of *p* < 0.05 was considered statistically significant. Statistical analysis was performed using IBM SPSS Statistics (Version 29.0.1.1, 2023, IBM Corporation, New York, USA).

## Results

### Baseline characteristics of the overall study population

The study included 215 patients with a median age of 80 (IQR 7) years, 69% of which were female. Arterial hypertension (82.3%), atrial fibrillation (92%), and chronic kidney disease were frequent (50.7%). The median EuroSCORE II for the overall population was 4.02% (IQR 4.50) and the median CRP/albumin ratio (CAR) was 1 (IQR 1.693).

In a five-grade TR grading scheme, most patients had severe TR (III° of V°) (58%) and median left-ventricular ejection fraction (LV-EF) was 55% (IQR 7). Detailed baseline characteristics are summarized in Table 1.

**Table 1:** Baseline characteristics and echocardiographic parameters of the overall study population and the high and low CAR groups

Parameters	Overall (*n* = 215)	High CAR group (*n* = 93)	Low CAR group (*n* = 122)	*p* value
Age (years)	80 (7)	79 (7)	80 (7)	0.929
Female	149 (69.3)	61 (65.6)	88 (72)	0.303
BMI kg/m^2^	25.55 (6.94)	26.23 (5.94)	25.25 (7.4)	0.343
NYHA class III/IV	182 (84.7)	85 (91.4)	97 (79.5)	0.017*
6-MWT meters	276 (172)	208 (110)	294 (135)	< 0.001*
MLHFQ	40 (26)	47 (29)	35 (27)	0.007*
Peripheral edema	139 (79.9)	59 (85.5)	80 (76.2)	0.134
EuroSCORE II	4.02 (4.50)	4.82 (4.375)	3.69 (4.25)	0.011*
TRI-SCORE	5 (3)	6 (2.75)	4 (3)	< 0.001*
eGFR (ml/min/m^2^)	40 (24)	36 (22)	43.5 (21)	0.002*
Bilirubin (mg/dl)	0.7 (0.5)	0.7 (0.7)	0.7 (0.5)	0.676
NT-proBNP (ng/dl)	2093 (2743)	3013 (3513)	1681 (1703)	0.001*
CRP (mg/L)	3.1 (6.5)	10.3 (17.82)	1.8 (1.7)	< 0.001*
Albumin (g/l)	40 (6)	39 (5)	41 (6)	< 0.001*
CAR	1 (1.693)	2.406 (3.081)	0.481 (0.463)	0.001*
Arterial hypertension	177 (82.3)	73 (78.4)	104 (85.2)	0.199
Atrial fibrillation	198 (92.1)	86 (92.5)	112 (91.8)	0.857
Chronic kidney disease	109 (50.7)	55 (59.1)	54 (44.3)	0.031*
Coronary artery disease	88 (40.9)	41 (44.1)	47 (38.5)	0.411
Dialysis	6 (2.8)	3 (3.2)	3 (2.5)	1
Diabetes	53 (24.7)	29 (31.2)	24 (19.7)	0.052
Echocardiographic parameters				
LV-EF (%)	55 (7)	55 (11.50)	55 (8)	0.723
FAC (%)	34 (10)	30.5 (10.7)	36 (9)	0.007*
TAPSE (mm)	17 (6)	17 (6)	18 (6)	0.333
EROA (cm^2^)	0.61 (0.44)	0.58 (0.57)	0.61 (0.38)	0.544
Vena contracta (mm)	12 (6)	12 (5.75)	12 (6)	0.759
Regurgitant volume (ml)	46 (26.2)	47 (25.5)	46 (28)	0.886
Right atrial area (cm^2^)	33 (14)	31 (16)	34 (13)	0.198
RV basal diameter (mm)	44 (8)	44 (7.5)	44 (7)	0.226
Vena cava diameter (mm)	25 (6)	26 (8)	24 (6)	0.630
TR grade				
Severe (III)	122 (58.1)	57 (63.3)	65 (54.2)	
Massive (IV)	52 (24.8)	16 (17.8)	36 (30)	
Torrential (V)	36 (17.1)	17 (18.9)	19 (15.8)	

Values are median (*IQR* interquartile range) or *n* number of patients (%). Percentages refer to eligible patients

*BMI* body mass index, *NYHA* New York Heart Association functional class, *6-MWT* 6-min walk test, *MLHFQ* Minnesota Living with Heart Failure Questionnaire, *LV*-*EF* left-ventricular ejection fraction, *FAC* right-ventricular fractional area shortening, *TAPSE* tricuspid annular plane excursion, *TR* tricuspid regurgitation, *EROA* effective regurgitation orifice area, *RV* right ventricle, *eGFR* estimated glomerular filtration rate, *CRP* C-reactive protein, *CAR* CRP/albumin ratio

^*^Statistically significant (Mann–Whitney *U* test or Pearson Chi-square test, *p* < 0.05)

### Procedural characteristics and clinical outcomes of the overall study population

**Fig. 1:**
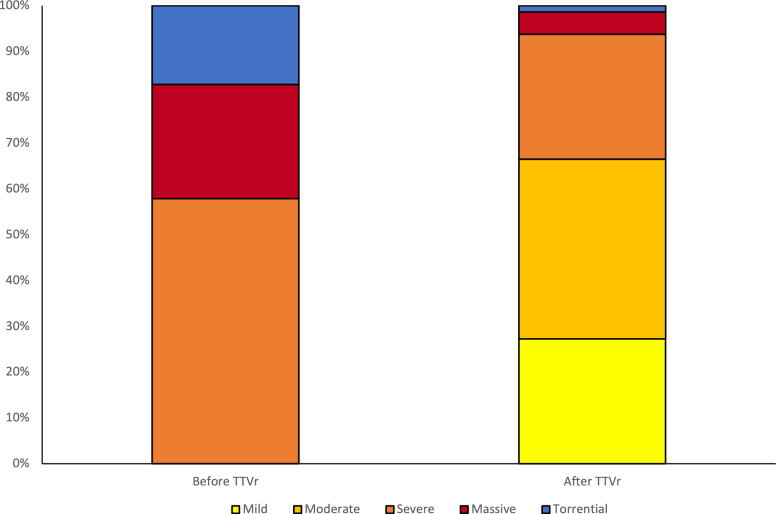
Percentages of tricuspid regurgitation (TR) grades before and after transcatheter tricuspid valve repair (TTVr) in the overall study population

In the overall study population, not stratified by CAR, most patients were treated by T-TEER (62%), whereas 38% were treated with TTVA. Median right atrial pressure was elevated (17 mmHg) and intraprocedural success was achieved in 61% of patients. Patients with intraprocedural success had significantly smaller median RV basal diameters (43 mm vs 47 mm, *p* < 0.001) and median right atrial area (32cm^2^ vs 35cm^2^, *p* = 0.022) compared to patients with procedural failure. Figure 1 shows the frequency distribution of TR grades before and after TTVr. In the overall population 51% of patients experienced an improvement of NYHA functional class after TTVr at 30 day follow-up. Median distance in the 6-MWT improved by 17.5 (IQR 87) meters and the MLHFQ was reduced by 12 points (IQR 15) at 30 days. 22.7% of patients were hospitalized for heart failure during the 2 years of follow-up. In the overall study population, the primary endpoint of all-cause mortality occurred in 55 (27.6%) of patients in the 2 years after TTVr. There was no significant difference in occurrence of the primary endpoint between patients treated with T-TEER or TTVA (31 (24%) vs. 24 (33.8%), *p* = 0.264, log-rank test). Further findings are summarized in Table 2.

**Table 2:** Procedural characteristics and outcomes and clinical outcomes of the overall population and the CAR groups

Parameter	Overall (*n* = 215)	High CAR group (*n* = 93)	Low CAR group (*n* = 122)	*p* value
Type of procedure				
Edge-to-edge-repair	134 (62.3)	62 (66.7)	72 (59)	0.251
Direct annuloplasty	81 (37.7)	31 (33.3)	50 (41)	0.251
Hemodynamic parameters				
Cardiac index (L/min/m^2^)	2.5 (1.3)	3 (1.3)	2 (0.5)	0.792
Cardiac output (L/min)	4.7 (2)	5.58 (2.2)	4.32 (1.2)	0.564
Mean right atrial pressure (mmHg)	17 (11)	19 (6)	12 (11)	0.013*
Mean pulmonary artery pressure (mmHg)	27 (13)	35 (11)	25 (6)	0.928
Pulmonary vascular resistance (wood units)	2.4 (1.7)	2.4 (1.9)	2.4 (1.7)	0.892
Procedural outcomes				
Intervention duration (minutes)	188 (77)	170 (68)	188 (95)	0.609
Hospitalization after intervention (days)	7 (5)	7 (7)	6 (4)	0.057
TR grade after intervention	2 (2)	3 (2)	1 (1)	0.074
Intraprocedural success (TVARC)	131 (61)	47 (50.5)	84 (68.9)	0.006*
Clinical outcomes				
NYHA class III/IV at 30 days (*N* = 183)	63 (32.3)	29 (35.4)	34 (30.1)	0.437
Improvement in NYHA class (1 or more) at 30 days (*N* = 183)	109 (50.7)	43 (39.4)	66 (60.6)	0.253
Change in 6-MWT meters at 30 days (*N* = 105)	17.5 (87)	21 (106)	14 (82)	0.823
Change in MLHFQ at 30 days (*N* = 112)	− 12 (15)	− 13 (16)	− 11 (14)	0.980
Heart failure hospitalization during follow-up (*N* = 185)	42 (22.7)	22 (29.3)	20 (18.2)	0.079
All-cause death during follow-up (*N* = 215)	55 (27.6)	38 (43.4)	17 (15)	< 0.001*

Values are median (*IQR* interquartile range) or *n* number of patients (%). Percentages refer to eligible patients

*N* number of patients with available data, *CAR* CRP/albumin ratio, *NYHA* New York Heart Association functional class, *6-MWT* 6-min walk test, *MLHFQ* Minnesota Living with Heart Failure Questionnaire, *TR* tricuspid regurgitation, *TVARC* Tricuspid Valve Academic Research Consortium

^*^Significant difference between CAR groups (*p* < 0.05 Mann–Whitney *U* test, Pearson Chi-square test or log-rank test)

### Assessing CAR for prediction of mortality after transcatheter tricuspid valve repair and calculation of the optimal CAR threshold

**Fig. 2:**
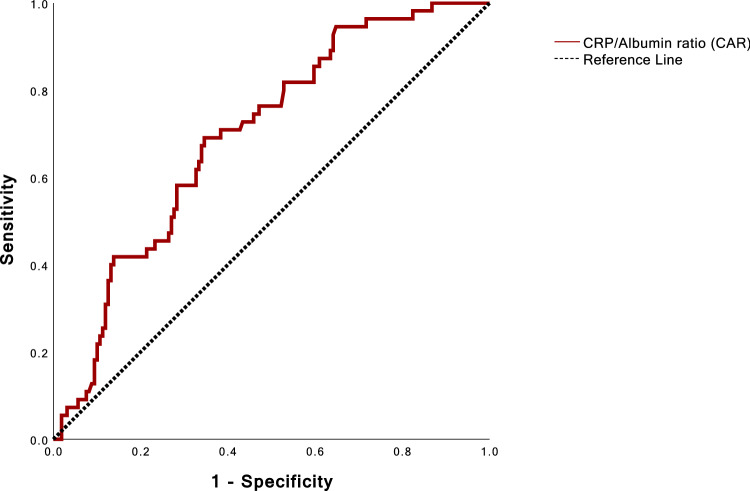
Receiver-operating characteristics curve for the CRP/albumin ratio predicting death after interventional tricuspid valve repair. Area under the curve was 0.695

AUC of CAR was 0.695 (Fig. 2). AUC of CRP was 0.692 and AUC of albumin was 0.640 individually. The optimal threshold and cut-off with the best trade-off between sensitivity and specificity was found at a CAR of 1.2945. The cut-off of 1.2945 was used to divide the patients in two groups: the high CAR group (CAR >  = 1.2945, *n* = 93) and the low CAR group (CAR < 1.2945, *n* = 122).

### Baseline characteristics, procedural, and clinical outcomes of the CAR groups

Comparing the two CAR groups, the high CAR group had significantly worse renal function indicated by lower median estimated glomerular filtration rate (eGFR 36 vs 44 ml/min/m^2^). Patients in the high CAR group also had significantly shorter walking distance in 6-MWT and worse scoring on the MLHFQ at baseline (Tables 1 and 2). Invasive hemodynamic assessment revealed no significant difference in cardiac index or cardiac output, mean pulmonary artery pressure, or pulmonary vascular resistance. Mean right atrial pressure was significantly increased in the high CAR group (19 vs. 12 mmHg). While procedural data were similar in both groups, significantly less intraprocedural success was observed in the high CAR group (50 vs. 69%) (Table 2). Change in 6-MWT, MLHFQ, or improvement in NYHA class was not significantly different between both groups at 30 days (Table 2).

### CAR is independently associated with increased mortality during 2-year follow-up

**Fig. 3:**
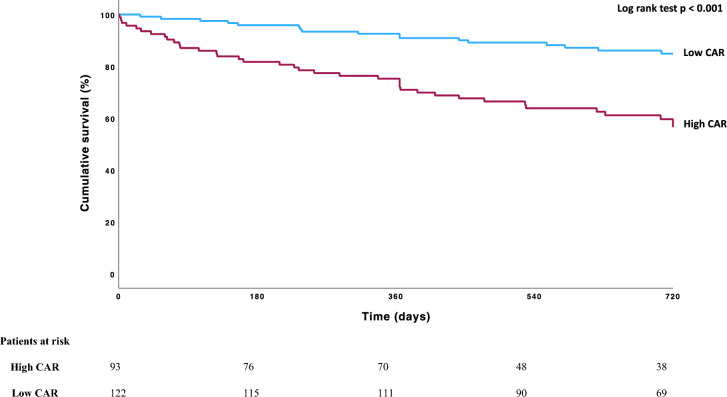
Kaplan–Meier curve of estimated survival comparing high and low CAR groups. Optimal Cut-off for CAR was 1.2945 (Youden-Index). Significantly higher mortality in the high CAR group (*p* < 0.001, log rank test). *CAR* CRP/Albumin ratio

A Kaplan–Meier plot was calculated for survival analysis, comparing the high and low CAR groups (Fig. 3). Early divergence of the survival curves could be observed. The primary endpoint occurred significantly more often in the high CAR group in the 2 years following TTVr (43.4%) compared to the low CAR group (15%, *p* < 0.001, Table 2).

Patients in the high CAR group had an increased mortality risk during the follow-up period (HR 3.512; 95% CI 1.981–6.226; *p* < 0.001). In multivariable analysis, this risk remained significantly increased when adjusted for factors tested significant in univariable analysis (eGFR, bilirubin, fractional area shortening (FAC), and procedural failure (HR 2.188; 95% CI 1.198–3.998; *p* = 0.011) (Table 3). This independent association of high CAR also remained significant when excluding patients treated with TTVA from the analysis (HR 3.540; CI 95% 1.389–9.021, *p* = 0.008). CAR had a better predictive value (AUC) than the EuroSCORE II and was only outperformed by the multi-parametric TRI-SCORE (Supplementary results). In an exploratory analysis, CAR was combined with the TRI-SCORE improving its AUC (Suppl. Figure 1, Supplementary results). In an exploratory analysis, we identified 18 (8.4%) of our patients having a CAR over 6.85. These very high CAR patients had a limited estimated survival of only 50% at 1-year follow-up after TTVr.

**Table 3:** Univariable and multivariable Cox regression analyses for risk of death during follow-up

Parameter	Univariable HR (CI 95%)	*p* value	Multivariable HR (CI 95%)	*p* value
High CAR vs low CAR	3.512 (1.981–6.226)	< 0.001*	2.188 (1.198–3.998)	0.011*
Age per year	0.987 (0.959–1.016)	0.372		
Male gender	1.524 (0.884–2.6379	0.129		
BMI per kg/m^2^	0.992 (0.946–1.039)	0.730		
eGFR	0.982 (0.966–0.999)	0.034*	0.993 (0.978–1.010)	0.424
Bilirubin	1.872 (1.387–2.535)	< 0.001*	1.822 (1.296–2.560)	< 0.001*
CRP	1.007 (0.997–1.017)	0.174		
Coronary artery disease	1.014 (0.593–1.734)	0.959		
Arterial hypertension	0.691 (0.371–1.288)	0.245		
Diabetes	1.741 (0.998–3.035)	0.051		
Chronic kidney disease (eGFR < 60 ml/min%)	1.575 (0.918–2.702)	0.099		
Dialysis	1.439 (0.351–5.907)	0.613		
Atrial fibrillation	1.710 (0.732–3.996)	0.215		
LV-EF per %	0.984 (0.959–1.010)	0.221		
TR grade	1.197 (0.866–1.655)	0.276		
FAC per %	0.954 (0.927–0.982)	0.001*	0.966 (0.938–0.995)	0.023*
Procedural failure (TVARC)	3.329 (1.921–5.769)	< 0.001*	2.759 (1.540–4.941)	< 0.001*

Risk of death during follow-up in the high CAR group after co-adjustment for other predictors significant in the univariable analysis

*BMI* Body mass index, *CRP* C-reactive protein, *CAR* CRP/Albumin ratio, *eGFR* estimated glomerular filtration rate, *LV-EF* left-ventricular ejection fraction, *TR* tricuspid regurgitation, *FAC* fractional area shortening, *NYHA* New York Heart Association functional class, *TVARC* Tricuspid Valve Academic Research Consortium

^*^Statistically significant (*p* < 0.05, Cox regression)

### Impact of intraprocedural success on symptomatic and clinical benefit in patients with high and low CAR

**Fig. 4:**
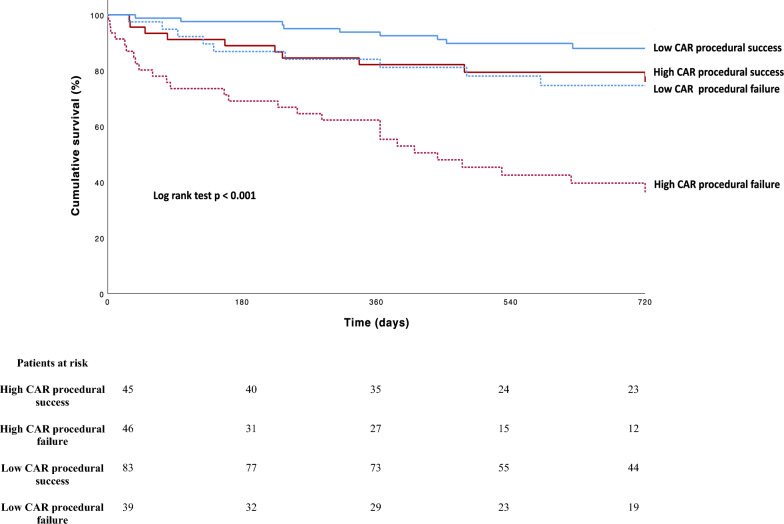
Kaplan–Meier curve of estimated survival comparing high and low CAR groups with and without intraprocedural success according to TVARC. Optimal cut-off for CAR was 1.2945 (Youden Index). Significantly higher mortality in the high CAR group with procedural failure (*p* < 0.001, log-rank test). *CAR* CRP/Albumin ratio, *TVARC* Tricuspid Valve Academic Research Consortium

The CAR groups were separately assessed for symptomatic and clinical benefit after TTVr according to intraprocedural success. No significant difference in improvement of symptom burden (6-MWT, MLHFQ, NYHA functional class) at 30 days was observed in high CAR group patients undergoing a procedure with intraprocedural success according to TVARC criteria. The primary endpoint of death occurred significantly less during follow-up in patients of the high CAR group with intraprocedural success (26.6% vs 60.5%, *p* < 0.001). Figure 4 delineates the survival curves of the CAR groups stratified by intraprocedural success in Kaplan–Meier analysis.

In patients of the low CAR group, no significant improvements in mortality or symptom burden were observed in the group with intraprocedural success (Table 4).

**Table 4:** Treatment benefit in patients with high versus low CAR stratified by procedural success

Groups	Outcome	Procedural success (TVARC) (total *n* = 131)	Procedural failure (TVARC) (total *n* = 84)	*p* value
High CAR (total *n* = 93)		*n* = 47	*n* = 46	
	All-cause death during follow-up	11 (23%)	27 (59%)	< 0.001*
	Improvement in NYHA class (1 or more) at 30 days	21 (44.7%)	17 (37%)	0.76
	Change in 6-MWT meters at 30 days (*N* = 36)	21 (130)	14 (65)	0.973
	Change in MLHFQ at 30 days (*N* = 38)	− 13 (21)	− 11.50 (10)	0.676
Low CAR (total *n* = 122)		*n* = 84	*n* = 38	
	All-cause death during follow-up	9 (11%)	8 (21%)	0.098
	Improvement in NYHA class (1 or more) at 30 days	47 (56%)	19 (50%)	0.541
	Change in 6-MWT meters at 30 days (*N* = 69)	12 (76)	14 (88)	0.517
	Change in MLHFQ at 30 days (*N* = 74)	− 11 (12)	− 13 (25)	0.516

Values are median (*IQR* interquartile range) or *n* number of patients (%). *N* number of patients with available data. Percentages refer to eligible patients with either procedural success or failure for each CAR group

*CAR* CRP/albumin ratio. *NYHA* New York Heart Association functional class. *6-MWT* 6-min walk test, *MLHFQ* Minnesota Living with Heart Failure Questionnaire, *TVARC* Tricuspid Valve Academic Research Consortium, *IQR* interquartile range

^*^Significant difference between CAR groups (*p* < 0.05 Mann–Whitney *U* test, Pearson Chi-square test or log-rank test)

### Sensitivity analysis: evaluation of infections as a driver of mortality in the high CAR group

CRP is an inflammatory marker, which is also elevated in infections [10]. Sensitivity analysis to evaluate the role of infections in mortality in the high CAR group was performed. The cut-off for the 95th percentile of all CRP values was at 31.6 mg/dl. The highest 5% of CRP values had a median CRP level of 42.8 mg/dl (IQR 17 mg/dl), and all cases were allocated in the high CAR group. 181 mg/dl was the highest of all CRP levels.

Infection was documented in six (54%) cases of these CRP outliers. Most commonly urinary tract infections (Suppl. Table 1) were documented. In all cases, infection was detected after the procedure and three of these cases died during follow-up after more than 60 days after TTVr (Suppl. Table 1). CAR remained significantly and independently associated with mortality even after exclusion of the CRP outliers (HR 2.3; 95% CI (1.261–4.348), *p* = 0.007).

## Discussion

Aim of this study was to evaluate the association of CAR with mortality after TTVr. The main findings were:

- Patients of the high CAR subgroup had significantly worse renal function, increased mean right atrial pressure, and demonstrated less intraprocedural success compared to the low CAR group.
- CAR was independently associated with worse survival after TTVr even after adjusting for other significant parameters.
- Achieving intraprocedural success in patients with high CAR led to better survival after TTVr compared to patients with high CAR and procedural failure.

Risk stratification is key in identifying patients which benefit from TTVr. In search of everyday parameters representing risk in patients undergoing TTVr, we investigated CAR and its association with mortality. As a matter of fact, severe TR is complicated by backward failure and many patients present in late-stage disease [1]. Thus, CAR bears the potential of being readily available and reflects multi-organ complications of TR due to backward failure identifying high-risk patients. To the best of our knowledge, this is the first study to analyze CAR in the setting of TTVr.

The group of patients which died during follow-up had significantly higher CRP and lower albumin levels. Backward failure with intestinal congestion and micro-translocation of bacteria leading to systemic low-grade inflammation might be a mechanism of CRP elevation [6]. Advanced organ damage due to systemic congestion might further contribute to this inflammation and systemic congestion is represented by the higher right atrial pressure in the high CAR group. The overall study population was elderly and comorbidities were common, representing patients at risk for nosocomial infections [21]. As patients with pre-procedural infections were excluded or had TTVr postponed until clearance of infection, infections do not seem to be a relevant driver of mortality in the immediate postinterventional period in this study population. Patients with the highest CRP values were evaluated to assess infections as a cause of high CRP and driver of mortality. While infections were present in the patients with the highest CRP values, infections occurred only after procedure and were not associated with death in the first days after procedure. High CAR remained significantly associated with mortality even after exclusion of the CRP outliers, which supports that CAR reflects low-grade inflammation. The low albumin levels in the high CAR group can be explained by malabsorption and following malnutrition due to intestinal wall congestion [6]. Further hepatic congestion leads to hepatic damage and decreased hepatic synthesis of albumin [22]. Hepatic impairment is also represented by the significantly higher bilirubin in the high CAR group. Low albumin therefore also reflects backward organ damage due to TR.

Both CRP and albumin contribute to the CAR increase in this study, this being representative of progressive right-heart failure with systemic congestion, intestinal impairment with malnutrition, and low-grade systemic inflammation described by the other studies [6]. This study underlines this aspect of TR pathophysiology and suggests that increased attention should be given to patient factors like malnutrition and systemic low-grade inflammation representative of organ damage.

Several factors might drive the mortality in the high CAR group including low-grade inflammation, malnutrition, and organ damage which all can be attributed to systemic congestion. Increased right atrial pressures reflect the backward transmitted splanchnic congestion leading to multi-organ damage [6]. Mean right atrial pressure was increased in the high CAR group accompanied by decreased renal function. Studies have previously linked increased right atrial pressure with decreased renal function and increased mortality [5, 23].

Increasing evidence suggests that patients in late-stage RV failure due to TR do not profit from TTVr and timing of intervention is essential [24, 25]. Increased right atrial pressures in the high CAR group might generally indicate decompensated patients at procedure and increased focus might be given to pre-procedural decongestion and optimization before procedure. On the other hand, there were no significant differences in peripheral edema at baseline between the CAR groups in this study, indicating clinical euvolemia. This might mean that persistently increased right atrial pressures after recompensation reflect more severe TR and atrial dysfunction in otherwise euvolemic patients.

High CAR was independently associated with increased mortality 2 years after TTVr after adjusting for RV function, renal and liver function, and importantly procedural failure according to TVARC criteria. The increased risk of death independent of the procedural outcome would suggest that patients with high-grade TR and late-stage RV failure might not benefit from TTVr, even if substantial TR reduction can be achieved [24, 25]. Opposingly, patients of the high CAR group with intraprocedural success had significantly reduced mortality during follow-up. This indicates that while patients with high CAR have higher mortality risk, they have a survival benefit when achieving intraprocedural success during TTVr. This effect was not observed in the low CAR group. This study therefore identifies a high-risk subgroup which may lower its mortality risk due to a successful TTVr procedure. On the other side, a very high CAR of 6.85 or more identified patients with a dismal prognosis of only 50% 1-year survival after TTVr. These results suggest a rather conservative approach in patients with very high CAR and careful consideration of other patient characteristics is warranted. However, multicenter studies are needed to generalize these findings.

CAR has been tested in other patient groups and conditions for predictive power of mortality or disease activity [15, 26, 27]. Increased CAR has been associated with increased risk of cardiovascular disease, and has been shown to be a marker for 30-day mortality after transcatheter aortic valve replacement [16, 28]. However, this is the first study evaluating CAR after tricuspid valve repair. Another study has already assessed the nutritional status using the mini nutritional assessment score including albumin as a prognosticator after TTVr [29]. Albumin was also included in a novel risk score predicting mortality in isolated TR [30]. However, these scores neglect low-grade inflammation, which is reflected by combining CRP with albumin in CAR. Further studies should characterize the low-grade inflammation induced by TR and investigate the impact of TTVr on this pathophysiological aspect. Finally, in our supplementary analysis, the TRI-SCORE showed a slightly better AUC than CAR. Compared to the multi-parametric TRI-SCORE, the components of CAR are routinely available in everyday practice and easily calculated. Since adding CAR to the TRI-SCORE in the supplementary analysis led to an increase in predictive capacity, this could be an option to use CAR in addition to established risk scores. The novel multi-parametric TRIVALVE Score predicts mortality and rehospitalization in the 12 months after TTVr, [31] It should be noted that the multi-parametric TRIVALVE score had a slightly worse AUC (0.681) than CAR. However, a direct comparison to CAR is limited due to divergent endpoints and future studies could analyze the combination of CAR and TRIVALVE after TTVr.

### Limitations

Limitations of this study include the retrospective and single-center design. Comparative studies evaluating the TRI-SCORE from other centers showed even more morbid patients which were treated by TTVr compared to this study population, and this might influence the generalizability of scores like CAR [4]. Furthermore, this study included a population treated with either T-TEER or TTVA, representing different interventional techniques. However, there was no significant difference between the different types of procedures regarding mortality during follow-up. High sensitive CRP (hs-CRP) is able to detect even lower levels of inflammation and is associated with cardiovascular events and mortality [32]. In this study, only CRP, as a routinely available marker of inflammation, was investigated. Although hs-CRP is more costly, it might improve the sensitivity of CAR and could be investigated in further studies.

## Conclusion

CAR has the potential to predict mortality after TTVr. This marker might identify patients with progressive right-heart failure with systemic inflammation, malnutrition, and organ damage. Further, CAR might identify patients which have an especially high mortality risk after an unsuccessful TTVr procedure. CAR is readily available in clinical routine and might be used in adjunction to establish risk scores, but further investigations are needed to explore its full value in populations undergoing valvular interventions.

## Supplementary Information

Below is the link to the electronic supplementary material.

Supplementary file1 (DOCX 28 KB)

## Abbreviations

6-MWT: 6-Minute walk test
CAR: CRP/albumin ratio
CRP: C-reactive protein
eGFR: Estimated glomerular filtration rate
EROA: Effective regurgitant orifice area
FAC: Fractional area shortening
MLHFQ: Minnesota Living with Heart Failure Questionnaire
NYHA: New York Heart Association functional class
RV: Right ventricle
TTVA: Transcatheter direct annuloplasty
T-TEER: Transcatheter edge-to-edge repair
TR: Tricuspid regurgitation
TTVr: Transcatheter tricuspid valve repair
TVARC: Tricuspid Valve Academic Research Consortium
