# Supplementary material for: C-reactive protein-to-albumin ratio is associated with mortality after transcatheter tricuspid valve repair
Source: Clin Res Cardiol. 2025 Apr 10;114(7):892–903. doi: 10.1007/s00392-025-02641-4 (PMC12202701; doi:10.1007/s00392-025-02641-4)
Supplement: Supplementary file 1 — Supplementary file1 (DOCX 28 KB) [file 392_2025_2641_MOESM1_ESM.docx]

**C-reactive Protein to Albumin ratio is associated with Mortality after Transcatheter Tricuspid Valve Repair**

# Supplementary material -

**Authors:**

Karl Finke^1^*, Laura Marx^1^, Jan Althoff^1^, Thorsten Gietzen^1^, Matthieu Schäfer^1^, Jan Wrobel^1^, Philipp von Stein^1,2^, Jennifer von Stein^1,2^, Maria Isabel Körber^1^, Stephan Baldus^1^, Roman Pfister^1^, Christos Iliadis^1^

**Affiliations:**

^1^ University of Cologne, Faculty of Medicine and University Hospital Cologne, Department III of Internal Medicine, Cologne, Germany

^2^ Cardiovascular Research Foundation, New York, New York.

**Supplementary table 1: CRP-outliers and rates of infection, antibiotic treatment, and microbiological findings.**

| Patient | CRP mg/dl  before procedure | High CAR group (1 = yes, 0 = no) | Documented infection (1 = yes, 0 = no) | Antibiotic treatment (1 = yes, 0 = no) | Microbiological finding (1 = yes, 0 = no) | Death during follow-up |
| --- | --- | --- | --- | --- | --- | --- |
| 1 | 52 | 1 | 1 | 1 | 0 | 0 |
| 2 | 53 | 1 | 0 | 0 | 0 | 1 |
| 3 | 41 | 1 | 0 | 0 | 0 | 0 |
| 4 | 35 | 1 | 0 | 0 | 0 | 0 |
| 5 | 32 | 1 | 1 | 1 | 0 | 0 |
| 6 | 48 | 1 | 1 | 1 | 1 | 1 |
| 7 | 46 | 1 | 1 | 1 | 1 | 1 |
| 8 | 181 | 1 | 1 | 1 | 0 | 0 |
| 9 | 43 | 1 | 1 | 1 | 1 | 1 |
| 10 | 39 | 1 | 0 | 0 | 0 | 0 |
| 11 | 32 | 1 | 0 | 0 | 0 | 1 |

*CRP = C-reactive protein, CAR = CRP/albumin ratio.*

**Supplementary figure 1: Receiver operating characteristics (ROC) curves of the CRP/albumin ratio (CAR), EuroSCORE II, TRI-SCORE and the TRI-SCORE + CAR for prediction of mortality after interventional tricuspid valve repair.**

**Supplementary results:**

**The predictive power of the CAR compared to the EuroSCORE II and TRI-SCORE**

The CAR was associated with increased mortality in the 720 days after TTVr. For evaluation of the performance of CAR compared to other established risk scores the ROC curves of EuroSCORE II and the TRI-SCORE were calculated. (Supplementary figure 1). CAR had a higher AUC compared to the EuroSCORE II (0.695 CI 95 % 0.618 – 0.771 vs 0.654 CI 95 % 0.569 – 0.739) indicating at least similar predictive performance. The TRI-SCORE had a slightly higher AUC than the CAR (AUC 0.750 CI 95 % 0.675 – 0.826 vs 0.695 CI 95 % 0.618 – 0.771). Adding the CAR as a new variable and one extra point to the multiparametric 12-point TRI-SCORE (TRI-SCORE + CAR) improved its AUC from 0.750 (CI 95 % CI 95 % 0.675 – 0.826) to 0.765 (CI 95 % 0.695 – 0.839).
